# The Role of Silver and Silver-Based Products in Wound Management: A Review of Advances and Current Landscape

**DOI:** 10.3390/jfb17010027

**Published:** 2026-01-01

**Authors:** Yiyao Du, Jianyu Lu, Xinya Guo, Zhaofan Xia, Shizhao Ji

**Affiliations:** Department of Burn Surgery, The First Affiliated Hospital of Naval Medical University, Shanghai 200433, China; duyiyao1127@126.com (Y.D.); 18796246278@163.com (J.L.); guoxinya0724@163.com (X.G.)

**Keywords:** antibacterial, silver-based materials, wound infection

## Abstract

The urgent need for silver-based antibacterial agents in clinical settings has driven the diversification of their delivery systems, evolving from traditional silver salt preparations to new silver nanoparticles (AgNPs) and silver-based composite functional materials. Research and application of various carrier systems have established a solid foundation for the clinical translation of silver. However, it is important to recognize that the clinical use of silver-based materials still faces several key challenges: one is the potential risk of cytotoxicity, another is the growing trend of bacterial resistance to silver, and the third is the heterogeneity of antibacterial properties in different wound microenvironments. Additionally, this study thoroughly examines the significant gap between basic research and clinical application of silver-based materials, highlighting that the lack of standardized clinical endpoint indicators and high-quality clinical research evidence are the main barriers to its standardized use. Future research should focus on four key areas: developing precise targeted delivery systems, creating combined treatments with silver and other antibacterial agents, enhancing biosafety through material engineering, and establishing a unified framework for clinical efficacy evaluation. Through systematic innovation and evidence-based clinical implementation, silver-based technologies hold broad potential and significant clinical value for addressing complex wound infections and alleviating the global antibiotic resistance crisis.

## 1. Introduction

Silver has been used in medicine as an antimicrobial agent for over a thousand years. The discovery and identification of bacteria and the development of microbial pathogenic theory in the early 19th century provided scientific support for the use of silver [1]. Silver salt products, such as silver sulfadiazine, have been used since then to treat burns and other wounds [2]. At the start of the 21st century, as traditional antibiotics faced growing drug resistance, researchers revisited silver as a potential antibacterial agent [3,4]. Meanwhile, advancements in materials science significantly transformed the applications of silver, making them more diverse. The rise of nanomaterials has revitalized silver’s applications, with silver nanoparticles (AgNPs), as potent antibacterial agents, beginning to be widely used in medical treatments [5,6]. In addition, silver ions (Ag^+^)and other polymer composites are also recognized by the medical community for their high biocompatibility and sustained antibacterial effect [7,8].

Silver-containing anti-infective products have significant advantages over traditional antibiotics. Firstly, silver has broad-spectrum antimicrobial properties, demonstrating resistance to a wide range of bacteria, fungi, and even viruses, including highly virulent, drug-resistant strains. Additionally, it inhibits bacterial biofilms that are highly resistant to existing antimicrobials, making it even more advantageous in the treatment of complex infections [9,10,11]. Secondly, silver is less likely to produce drug resistance, compared with antibiotics with a single mechanism. Silver has a complex and diverse antimicrobial mechanism, and its use can be a good solution to the problem of drug resistance brought about by the misuse of antibiotics. In addition, silver has a higher degree of stability, as silver is an inorganic material; it is subject to less influence by environmental factors, such as pH, temperature, enzymes, and other factors, and thus it can play a long-lasting role in the effect [12]. Most importantly, studies have shown that the combined use of silver and other antibiotics can produce a synergistic effect, which enhances the antimicrobial effect and expands the antimicrobial spectrum. Moreover, it achieves the purpose of reducing antibiotic use and minimizing the risk of drug resistance [13]. While possessing a strong antibacterial effect, silver has a low biotoxicity to the human body, and some studies have even shown that silver particles in a certain concentration range not only have low toxicity to the human body, but also can promote cell growth and tissue repair [9]. In a word, silver, as an inorganic element with significant antibacterial properties, provides a new strategy for the treatment of a variety of infectious diseases. Therefore, the research and development of silver is of indispensable value for promoting medical progress and improving human health.

**Figure 1:**
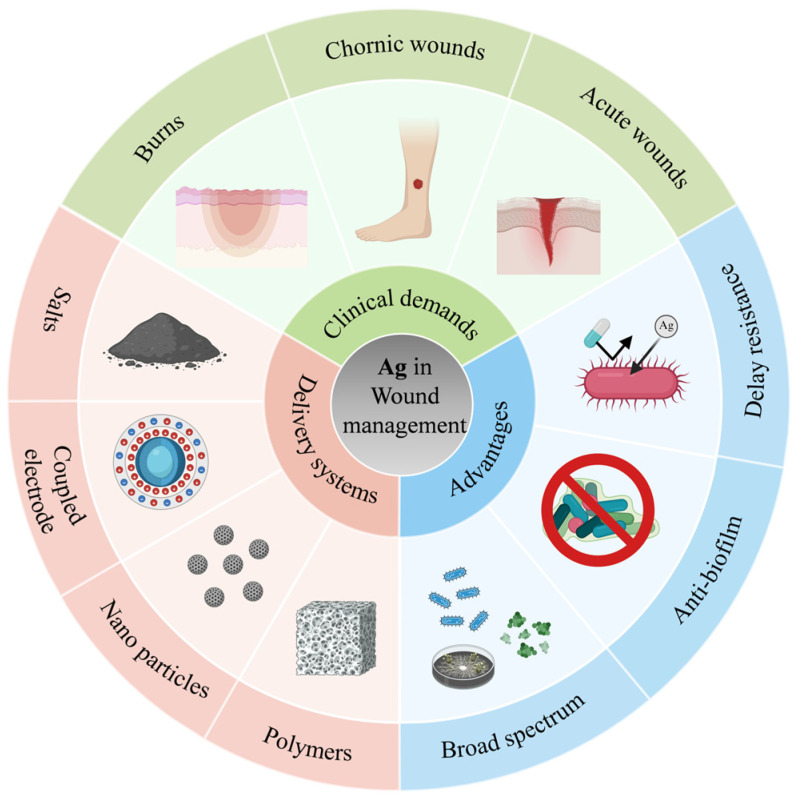
This review systematically examines the delivery systems of silver in wound management, along with the relevant clinical demands and requirements, and analyzes the comparative advantages of these approaches.

This review focuses on the clinical application of silver in various wound types, providing a brief examination of its research history and current status in wound management. It systematically summarizes the primary delivery systems, existing forms, advantages, and limitations of silver in anti-infective strategies—particularly regarding infection control and wound healing promotion across different wound etiologies. The article further summarizes the key molecular mechanisms underlying silver’s antimicrobial efficacy and provides a concise analysis of current application constraints. Accordingly, it proposes informed perspectives on future research directions and prospects for silver in medical practice. (Figure 1).

Both the long history of silver’s antimicrobial use and modern research clearly show its value and potential. However, despite the extensive clinical experience gathered over time, the fundamental principles of how it works are still not fully understood. With the rapid development of modern biology, chemistry, and materials science, understanding the precise mechanism of silver’s action at the molecular and cellular levels will not only unlock the secrets of its millennial effectiveness but also be the core driving force to optimize its application efficacy, expand its application scenarios, and ultimately promote the rational design of new-generation silver-based antimicrobial materials. Therefore, in-depth investigation of the antimicrobial mechanism of silver is the focus of current research.

## 2. Antimicrobial Mechanism

**Figure 2:**
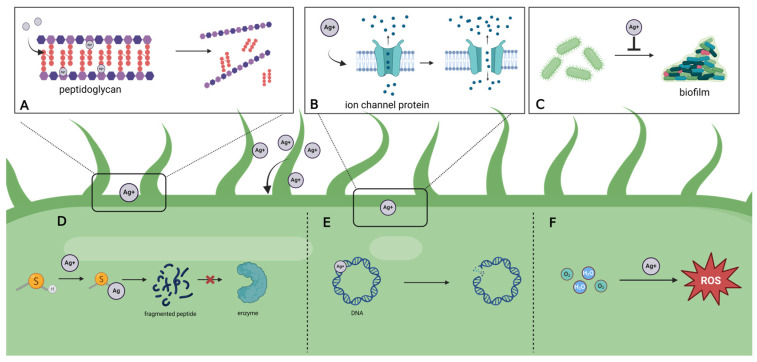
The antibacterial mechanisms of silver are as follows: (**A**). Binds to the peptidoglycan in bacterial cell walls, causing the breakage at the junction of peptide and glycan. (**B**). Strongly binds to ion channel proteins on bacterial cell membranes, impairing their function and leading to imbalances in intracellular and extracellular ion concentrations. (**C**). Inhibits biofilm formation. (**D**). Reacts with sulfhydryl groups, replacing hydrogen ions on these groups, preventing the formation of disulfide bonds during protein synthesis and further causing protein and enzyme inactivation. (**E**). Binds to DNA strands, leading to their breakage. (**F**). Induces oxidative stress, resulting in excessive ROS accumulation, which damages cells. This figure was created using BioRender (www.biorender.com).

The broad-spectrum antimicrobial efficacy of silver stems from its diverse antimicrobial mechanisms, which are mainly based on silver ions (Ag^+^) and silver nanoparticles (AgNPs). These mechanisms can be summarized into two main categories: direct and indirect effects. (Figure 2).

### 2.1. Direct Effects

Studies have shown that positively charged Ag^+^ can produce electrostatic adsorption effect with negatively charged bacterial membranes, tightly bind to lipoproteins or peptidoglycan on bacterial membranes and inhibit their functions, disrupting the selective permeability of cell membranes, leading to the disruption of ionic balance between inside and outside the bacterial membrane, for example, disruption of the concentration of important ions, such as potassium (K^+^), sodium (Na^+^), and calcium (Ca^+^), and thus causing bacterial death. (K^+^), sodium (Na^+^), calcium (Ca^+^), and other important ions, leading to bacterial death [14,15]. Ag^+^ can also inhibit the function of membrane proteins by binding to them, so that the function of the inner and outer membranes is damaged. Once Ag^+^ disrupts the outer membrane or cell wall of a bacterium, it can further penetrate the cell wall into the cell interior [16].

When silver enters the cell, it binds to DNA, ribosomes, and proteins of various biomolecules in the cell, destroying their normal biological functions and ultimately leading to the death of bacteria [17]. Recently, scholars found that Ag^+^ can cause DNA double-stranded dehybridization, weakening the binding of histone-like nuclear structure protein (H-NS) and DNA, accelerating the diffusion kinetics of H-NS protein, and impeding bacterial replication and translation [18]. In addition, Ag^+^ can inhibit normal biochemical functions by binding to enzymes and metabolic proteins in bacterial cells, leading to bacterial metabolic disorders. Ag^+^ can bind to sulfhydryl-containing enzymes, especially those related to energy metabolism, such as bacterial nitrate reductase A. The cationic reactivity property of Ag^+^ The cationic reactivity of Ag^+^ allows for it to attach to specific anionic regions and active sites of the three subunits of bacterial nitrate reductase A, inhibiting the enzyme activity and hindering the energy metabolism of the cell [19].

### 2.2. Indirect Effects

Ag^+^ induces ROS overproduction in bacteria in a time- and concentration-dependent manner, while inhibiting antioxidant defense systems such as catalase (CAT), peroxidase (POD), and reduced glutathione (GSH). This leads to a breakdown of redox homeostasis and ROS accumulation [20]. Excess ROS is capable of damaging membrane lipids, leading to membrane rupture, peroxidizing proteins, which can result in inactivation, peroxidizing DNA, causing DNA breaks or base damage, interrupting DNA replication and transcription, and ultimately leading to lethality [21]. Meanwhile, silver is effective against strongly resistant biofilms composed of bacterial populations and their secreted extracellular matrix components. The exact mechanism is not fully understood, and it is hypothesized that it may involve either Ag^+^ penetration of nutrient channels within the biofilm or direct penetration of AgNP into the biofilm matrix, making it uniquely advantageous in the treatment of biofilm infections, such as chronic wounds [22,23].

Despite these remarkable antimicrobial mechanisms of silver, the core challenge in translating these theoretical antimicrobial potentials into reliable efficacy in clinical practice is how to ensure that the silver acts on the target microorganisms “at the right time, at the right dose, and at the right site”. Therefore, the development of efficient, controllable, and safe silver delivery vehicles and technologies has become an essential bridge between antimicrobial mechanism research and practical applications. By designing different delivery platforms, it is possible to optimize the bioavailability of silver, prolong the duration of action, enhance the penetration of infection foci or biofilms, and mitigate the risk of systemic exposure.

## 3. Delivery Systems

Driven by continuous technological advancements, the modalities for delivering silver have become increasingly diverse. Current commonly employed delivery systems encompass a broad spectrum ranging from traditional silver salts to contemporary silver nanoparticles (AgNPs), and further extending to various innovative forms derived from AgNPs, such as silver-coupled electrodes and silver-based composite materials. This spectrum of sophisticated delivery approaches serves to overcome the limitations associated with the direct application of silver, thereby maximizing its antibacterial efficacy. (Table 1).

### 3.1. Silver Salts

Silver salts represent one of the earliest delivery forms of silver in the field of anti-infection, with their antibacterial effects primarily relying on the release of Ag^+^ ions. Currently, clinically common silver salts include silver nitrate, silver sulfadiazine, silver acetate, and silver chloride. In recent years, advancements in Ag^+^-based formulations have primarily been reflected in the development of combination therapy strategies.

Current research demonstrates that the co-administration of strontium acetate (SrAc) with silver nitrate (AgNO_3_) not only potentiates antimicrobial efficacy but also exhibits distinct osteoinductive properties, thereby presenting a novel therapeutic strategy for the prevention and management of peri-implantitis [41]. Furthermore, the silver nitrate/potassium tellurite (Ag/Te) complex has been clinically validated as among the most potent anti-biofilm agents against Pseudomonas aeruginosa. Of particular significance, this combination maintains remarkable bactericidal activity against clinically isolated strains exhibiting resistance to conventional antibiotics (e.g., gentamicin) and silver-based agents [42]. The Ag-Te combined therapeutic regimen exhibits three key advantages over traditional antibiotics: enhanced antimicrobial potency at lower concentrations, reduced bacterial regenerative capacity, and a mitigated propensity for resistance development. Mechanistic studies suggest that the synergistic antimicrobial effects arise through multimodal pathways, including regulation of thiol-redox homeostasis, induction of ROS (reactive oxygen species)-mediated oxidative stress, disruption of bacterial energy transduction systems, and permeabilization of microbial membrane integrity, while concomitantly augmenting the host’s endogenous antioxidant defenses [43].

Emerging evidence indicates that silver nitrate exhibits remarkable combinatory potential with antibiotics. Notably, the silver-gentamicin combination demonstrates pronounced synergistic activity against both Gram-positive and Gram-negative bacterial strains. Clinically, the incorporation of subtherapeutic gentamicin doses with silver nitrate into bone cement matrices has emerged as a cost-effective and therapeutically efficient approach for managing orthopedic infections [44]. From a materials science perspective, silver nitrate serves dual functions: beyond its direct antimicrobial utility, it acts as an essential precursor for the fabrication of silver nanoparticles (AgNPs) [45]. Through physicochemical and biological reduction pathways, Ag^+^ ions undergo controlled transformation into AgNP structures, thereby substantially broadening their application spectrum in biomedical engineering and related disciplines [46]. The synergistic effect of complex silver salts, such as Keggin-type heteropolyacid silver salts (Ag-HPA salts), in combination with itraconazole and amphotericin B, offers advantages including low cost, high yield, and potent activity at low concentrations [27].

Silver nitrate-based formulations maintain their preeminent position among silver-containing antimicrobial agents due to three cardinal advantages: cost-effectiveness, straightforward synthesis protocols, and extensive clinical validation. These factors collectively sustain their widespread clinical utilization as a cornerstone therapeutic option in antimicrobial management.

### 3.2. Silver Coupled Electrodes

Bioelectrical phenomena refer to the electrical manifestations that arise from biological activities. Bacteria primarily exhibit these effects through electron transfer and the generation of membrane potential gradients during metabolic processes. Current evidence confirms that leveraging bacterial bioelectricity enables growth inhibition via three principal mechanisms: alteration of membrane permeability, disruption of respiratory functions, and interference with energy metabolism [47]. Crucially, this approach also impairs biofilm-forming capacity, thereby reducing antibiotic resistance development [28]. Silver’s exceptional electrical conductivity enables its integration into coupled electrode systems designed to disrupt bacterial bioelectrical activity. Electrical stimulation not only enhances the susceptibility of biofilms to metal ions like Ag^+^ but also significantly increases antibacterial efficacy through synergistic interactions between electric fields and Ag^+^ ions [48].

Researchers have developed a wireless electroceutical dressing (WED) by integrating silver-zinc coupled electrodes into textile substrates. This polyester-based dressing features geometrically printed matrices of elemental silver and zinc particles. Upon contact with moist environments (e.g., wound exudate or hydrogel electrolytes), the dressing generates a low-intensity electric field (~1 V), inducing transient, micromolar-level superoxide anion production that effectively inhibits bacterial growth. Clinical trials demonstrated significantly reduced biofilm presence in WED-treated wounds (52% showing near-undetectable levels) compared to standard-of-care (SoC) groups (24% with low biofilm occurrence). The treatment also exhibited potent antibacterial effects against specific pathogens without compromising long-term wound healing outcomes. Currently FDA-cleared (510(k)#K180533) for both prescription and OTC use, WED has achieved commercialization in multiple countries beyond the United States [31].

An innovative wound dressing incorporating silver-graphene conductive cellulose composite has been developed through a distinctive biohybrid fabrication approach. This technology involves immobilizing graphene oxide (GO) onto bacterial cellulose substrates, followed by introducing polydopamine-coated silver nanoparticles (AgNPs) into Acetobacter culture medium to form composite membranes via biologically guided self-assembly. The resulting electrically conductive material generates therapeutic microcurrents and mild thermal effects in response to external voltage stimulation, significantly enhancing cell migration at the wound site. Simultaneously, the firmly anchored AgNPs on the material surface continuously release Ag^+^ ions while producing reactive oxygen species, thereby achieving a synergistic dual functionality that combines potent antibacterial action with tissue regeneration [32]. Coupling electrodes can also be applied to surgical implants via coating technology. For instance, a semi-transparent, robust, and conductive PEDOT-MeOH: PSS coating can be prepared through electrochemical polymerization of EDOT-MeOH and PSS. Subsequent aminosilane functionalization and chelation reactions immobilize Ag^+^ ions, yielding an AgNP-PEDOT-MeOH: PSS composite conductive coating. Experimental results demonstrate that under stable electrical stimulation, this conductive coating nearly completely inhibits bacterial colonization and significantly reduces biofilm formation, exhibiting substantially superior antibacterial efficacy compared to standalone silver coatings or conjugated polymer coatings alone [30]. By applying mechanical stimulation to the piezoelectric polymer poly(vinylidene fluoride-trifluoroethylene) (PVDF-TrFE) through a lab-made mechano-bioreactor, an electrical microenvironment can be generated. The resulting electrical response of the material is then transmitted to bacterial cells (such as *Escherichia coli* and *Staphylococcus epidermidis*). Combined with silver nanoparticles (AgNPs) and the unique morphological characteristics of the material, this can induce significant antibacterial and anti-biofilm activity [29].

Silver-coupled electrodes represent a relatively novel class of antimicrobial materials with distinct advantages over conventional alternatives. These systems offer precisely controllable antibacterial effects, as Ag^+^ release occurs through electrode ionization with adjustable kinetics and concentration via modulation of electrical parameters such as potential and current. Furthermore, their antimicrobial performance exhibits greater stability against environmental interference compared to traditional silver-based materials [49]. In contrast to the relatively lower stability of Ag^+^ solutions and AgNPs, which tend to react with other substances and exhibit diminished antibacterial efficacy, silver-coupled electrodes demonstrate prolonged antimicrobial functionality. Research indicates that while silver ionization release decreases in multi-metal electrolytes compared to single-silver systems, it remains sufficient for effective antibacterial activity with extended ionization duration. This suggests that silver-zinc or silver-copper ionization systems may offer more sustained antimicrobial effects than pure silver electrodes while significantly reducing biological toxicity [50].

### 3.3. AgNP

Silver nanoparticles (AgNPs) demonstrate markedly superior antimicrobial efficacy compared to conventional silver formulations (e.g., silver salts or metallic silver particles). Defined as nanoscale materials with characteristic dimensions between 1 and 100 nm, AgNPs exhibit unique physicochemical properties that have enabled their expanding utilization across diverse biomedical applications [51].

Silver nanoparticles (AgNPs) exhibit superior antimicrobial properties attributable to their nanoscale dimensions. The minimal particle size confers a high surface-area-to-volume ratio, facilitating extensive bacterial contact and enhanced Ag^+^ release. This compact architecture further enables exceptional penetration capability, particularly effective in disrupting bacterial biofilms and penetrating cellular structures [52]. Critically, AgNPs offer tunable antimicrobial performance through controlled modulation of particle size, morphology, and surface functionalization, demonstrating significant design flexibility [53]. Research consistently establishes direct correlations between the antibacterial efficacy of AgNPs and their physical characteristics. Smaller diameters intensify antimicrobial effects through improved cell wall adhesion, membrane permeability disruption, and intracellular penetration. Meanwhile distinct morphological configurations (spherical, cubic, triangular) differentially mediate bacterial interactions to optimize bactericidal outcomes [54].

#### 3.3.1. Preparation Method

The traditional chemical synthesis of silver nanoparticles (AgNPs) often relies on multi-step, reagent-mediated processes. A typical procedure involves mixing silver nitrate with trisodium citrate in a water bath, followed by the addition of sodium borohydride as a reducing agent to generate AgNPs [55]. Finally, polyvinylpyrrolidone (PVP) capping is required to enhance stability—a step that is crucial because nanoparticles readily interact with proteins in biological environments to form a protein corona, which can affect the sterilization efficacy of AgNPs. Thus, capping treatment is crucial to ensuring the stability of their application [56,57].

Green synthesis has emerged as a current research hotspot due to its alignment with eco-friendly and biocompatibility requirements [58]. It is characterized by the core features of “requiring no additional stabilizers or capping agents and being environmentally friendly,” relying on natural biomolecules or green energy to mediate the reduction in metal ions. Regarding the mediation systems, plant-derived biomolecules are commonly used as green reducing agents. For example, AgNPs can be synthesized using green tea leaf extract as a natural reducing agent via microwave irradiation technology [59]. Alternatively, adding silver nitrate to an aqueous extract of Datura stramonium leaves and heating at pH 7 and 60 °C for 30 min can be used to prepare AgNPs [60]. Plant polyphenolic compounds, such as tannic acid, have also successfully mediated the synthesis of TA-AgNPs [61]. Fungi, on the other hand, can synthesize stable and morphologically controllable AgNPs by producing enzymes that serve dual functions as both reducing and capping agents [62]. Biomass materials, such as bacterial cellulose, also demonstrate dual functional advantages, acting as both reducing and capping agents to efficiently synthesize AgNPs under solar radiation induction [63].

Furthermore, green synthesis technologies also incorporate various novel auxiliary strategies: Solvent radiolysis can induce the generation of solvated electrons and reducing radicals, enabling the reduction in metal ions without the need for additional chemical reducing agents. This process can also produce sterile colloidal suspensions directly applicable to the medical field [64]. Electrochemical methods based on hemoglobin-modified boron-doped diamond electrodes allow for the green synthesis of colloidal silver in aqueous phases [65]. The laser ablation-assisted chemical reduction method can readily prepare silver-core/silicon-shell nanoparticle colloids, with particle sizes precisely tunable within the range of 2.5 to 6.3 nanometers by adjusting the water-ethanol ratio. The porous, amorphous silica shell not only significantly enhances the inertness and stability of the colloid but also results in lower nanoparticle toxicity compared to systems without silica coating, further expanding the biomedical application potential of AgNPs [66].

#### 3.3.2. Modification

Additionally, modification and functionalization have become a core research direction for further optimizing the biological activity, targeting capability, and biosafety of AgNPs. Various modification strategies employing functional molecules or nanomaterials have demonstrated significant advantages. Peptide modification is a crucial approach for enhancing the biological functions of AgNPs. Research has found that among the top 10 peptides (TOP10) with the highest synthesis activity for AgNPs, 9 contain EE and E[X]E structural motifs (E: Glutamic acid, X: any amino acid), providing a structural basis for screening peptide-based modifiers [67]. Modification with Myristoyl Tetrapeptide-6 (MT6) or Copper Tripeptide-1 (CuTP1) yields MT6-AgNP and CuTP1-AgNP conjugates. The wound closure rate for MT6-AgNP reached 71.97 ± 4.35%, which is approximately 5.48 times higher than that of free MT6 (*p* < 0.05). The wound closure rate for CuTP1-AgNP was 62.37 ± 18.33%, representing a 2.82-fold increase compared to free CuTP1 (*p* < 0.05) [35]. Modification with metals or metal oxides can enhance the antimicrobial performance and safety of AgNPs. For instance, AgNPs modified with CuO nanoparticles (NPs) exhibit significantly superior antibacterial effects compared to single metal ions [68]. The construction of bimetallic nanoparticles, such as Ag-Au or Ag-Cu, not only enhances antimicrobial activity but also reduces potential genotoxicity, thereby improving biosafety [69]. Furthermore, conjugate modification with biomolecules and antimicrobial agents enables functional synergy and targeting optimization. Silver nanoparticles (SN) prepared using green tea total polyphenol extract as a reductant, when conjugated with chlorhexidine (Cx), showed significantly enhanced antibacterial effects against *Staphylococcus aureus* and *Candida albicans* [70]. When homologous serum is used as a modifying agent, the presence of specific anti-infective antibodies within the serum can enhance the targeting ability of the nanocomposite. A typical example is the AgNP-Serum-18 conjugate system prepared using silver nitrate as the precursor, which provides a targeted strategy for treating infection-related diseases [71].

#### 3.3.3. Clinical Application

One of the primary directions for the clinical application of AgNPs is the development of coatings for medical substrates. By precisely controlling the preparation processes and properties of these coatings, synergistic optimization of antibacterial activity, biocompatibility, and ion release behavior can be achieved to meet clinical diagnostic and therapeutic needs. Regarding coating preparation, electrodeposition techniques are among the mainstream methods. For instance, direct current sputtering technology can be employed to deposit a silver layer directly onto titanium surfaces [72] Furthermore, electrochemical deposition techniques enable the controlled deposition of silver-containing calcium phosphate (Ag/Ca-P) coatings on surfaces with complex shapes and porous substrates. Notably, Ag/Ca-P coatings exhibit excellent osteoblast compatibility, providing a feasible modification strategy for orthopedic implant materials [73].

The microstructure and composition design of coatings directly influence their clinical application efficacy. The unique physicochemical properties of silver nanoparticles (AgNPs) enable the formation of dense, uniform coatings on surgical implants—constituting an active research focus in antimicrobial surface engineering [74]. Silver coatings modified with a porous structure exhibit unique ion-release characteristics: over time, the concentration of silver ions they release surpasses that of smooth coatings. For example, in porous coatings, silver nanoparticles are deposited deep within the aluminosilicate coating layer, whereas in smooth coatings, they are distributed on the surface. By the 96-h mark, the silver ion release concentrations reach (12.47 ± 1.11) μg/mL and (10.90 ± 1.51) μg/mL, respectively. Moreover, the antibacterial effect shows a statistically significant difference, with porous coatings outperforming smooth coatings, providing sustained action for infection prevention and control [34]. On the other hand, the design of Ag/Au nanoalloy coatings focuses on the intelligent regulation of ion release. This alloy releases very low levels of Ag^+^ under neutral physiological pH conditions, thereby reducing potential toxicity. In contrast, in the acidic microenvironment formed by bacterial biofilms, Ag^+^ release increases significantly, achieving “on-demand antibacterial” activity [75].

Since the beginning of the 21st century, AgNPs have represented the most significant advancement in silver-based anti-infective strategies, emerging as a central research focus in modern antimicrobial materials due to their superior antibacterial efficacy, structural engineerability, and multifunctionality. Nevertheless, AgNPs are more frequently utilized not as standalone agents but as synthetic foundations for developing sophisticated delivery platforms. This approach addresses complex clinical demands through the optimization of nanoscale architectures.

### 3.4. Silver-Based Polymers

Silver-based composite materials represent a novel class of biomedical materials that integrate the superior properties of silver with unique characteristics of other components, enabling tailored solutions for diverse clinical applications. These composites demonstrate remarkable potential across multiple medical domains, particularly in infection control, tissue regeneration, and multifunctional therapeutic platforms, by synergistically combining antimicrobial efficacy with enhanced material performance.

#### 3.4.1. Synthesis Method

The properties of silver-based polymers depend primarily on their microstructure, which is in turn determined by the synthesis strategy. The synthesis strategies for silver-based polymers are diverse. Based on the “binding and immobilization mechanisms of silver species with the polymer matrix,” common approaches can be summarized into four categories: coordination and supramolecular-driven assembly, covalent bonding, physical encapsulation and confinement, and in situ synthesis and integrated composite formation.

The coordination and supramolecular-driven assembly strategy relies on the dynamic coordination interactions or supramolecular self-assembly between silver ions and electron-donating groups (such as amino, carboxyl, and pyridyl groups) on polymer chains to achieve the immobilization of silver species and material formation. For instance, ethynyl disulfide ligands and silver salts can form coordination compounds ranging from one-dimensional to two-dimensional structures through coordination bonds; silver nitrate and guanosine monophosphate can be used to prepare metal–organic coordination polymer gels (Ag@GMP); and the cytosine-silver-cytosine (Cy-Ag-Cy) structure containing dynamic coordination bonds imparts pH and light responsiveness to polypropylene glycol polymers (Ag-Cy-PPG) [39,76]. Meanwhile, supramolecular strategies, such as polyoxometalate-triggered surface templating, can guide the assembly of tyrosine-based polymer-stabilized silver nanoparticles into membrane-bound vesicles and can be combined with RAFT polymerization technology to construct complex ternary nanohybrids [77].

Covalent chemical bonding involves the permanent anchoring of silver species, particularly AgNPs, onto polymer matrices or pre-functionalized surfaces through the formation of covalent bonds or strong chemical interactions. For example, thiol groups in cysteine-rich human hair keratin (HHK) can be used to in situ anchor silver nanoparticles onto nanofibers [40]. Alternatively, silver nanoparticles can be loaded onto crosslinked sites formed via the Petasis multi-component reaction on amine-functionalized Fe_3_O_4_ surfaces, producing Fe_3_O_4_@MIP/Ag composite materials [78].

Physical encapsulation and confinement focus on utilizing the steric hindrance, electrostatic interactions, or interfacial stabilization of polymers to achieve the encapsulation and localization of silver nanoparticles. This approach is relatively straightforward in process and easily integrated with existing fabrication technologies, with its key emphasis on addressing the agglomeration of AgNPs and controlling their spatial distribution. Established practices include encapsulating silver colloids using polyethyleneimine (PEI), polyvinylpyrrolidone (PVP), and block copolymers such as polyethylene oxide (e.g., PEO-*b*-P2VP PEO) and poly (2-vinylpyridine) (P2VP) [79]. Confining hydrophobic silver nanoparticles within the oil phase by coating bacteria with acrylate-functionalized polyethyleneimine to construct Pickering emulsions, followed by polymerization to obtain bacteria-imprinted beads (BIBs) [80]; and employing laser techniques (such as KrF excimer laser) o immobilize pre-synthesized silver nanoparticles onto polymer film surfaces physically, or modifying polyetheretherketone (PEEK) surfaces via laser-induced periodic surface structures (LIPSS) to enhance the anchoring of silver nanoparticles [81].

In situ synthesis and integrated composite formation involve the one-step reduction of silver precursors to Ag^+^ or AgNPs in the presence of polymers, combining material the formation of synchronous composites in a single process. This includes bio-inspired synthesis and synchronous composite formation during polymerization. The in situ approach yields composites with strong interfacial bonding and uniform dispersion. Examples include the hydrothermal synthesis of nanocomposites containing methyl methacrylate polymer and silver nanoparticles; rapid preparation of silver nanoparticle/chitosan-grafted polyvinyl alcohol hydrogels using microwave irradiation; synthesis of polymer–silver chloride nanocomposites via in situ nanoprecipitation; in situ synthesis of silver nanoparticles integrated into hydrogel dressings using modified guar gum polymer; and preparation of functional nanofiber membranes through polymer blending (e.g., ethyl cellulose/polyurethane) combined with surface coating polydopamine-assisted deposition of silver nanoparticles [59,82,83,84,85]. The use of green reducing agents such as polyphenols and polysaccharides not only reduces environmental toxicity but also often imparts inherent bioactivity to the materials, enabling multifunctional synergistic effects such as antioxidant activity and wound-healing promotion. For instance, silver nanoparticles (Ag@PL NPs) can be prepared using enzymatically phenolated lignin as both reducing and stabilizing agents, followed by coating onto composite surfaces; or biosynthesized silver nanoparticles using fungal biomass (Aspergillus fumigatus) can be combined with chitosan in a layered composite [86,87]. Additionally, enzymes or enzyme–polymer conjugates (e.g., CALB) can directly induce the formation of silver oxide nano-biohybrids in aqueous media [88].

#### 3.4.2. Multiple Biological Effects

In addition to excellent antibacterial efficacy, silver-based polymers also demonstrate other biological advantages in antimicrobial applications. On one hand, specific systems exhibit superior resistance to bacterial resistance under equivalent bacteriostatic conditions. For example, the coordination polymer formed between the cyano groups of graphitic carbon nitride (GCN) and silver nanoparticles (AgNPs) shows approximately 30 times greater effectiveness against AgNP-resistant bacteria compared to conventional AgNPs. Serial passaging experiments over 60 generations revealed that GCN/Ag induced only a mild increase in bacterial resistance, whereas conventional AgNPs triggered significant resistance by the 20th generation [38]. On the other hand, selective bacterial capture and killing have been demonstrated in experimental applications such as AgNP-embedded bacterial-imprinted polymer microbeads [80]. Furthermore, some polymers not only exert antibacterial effects but also promote wound repair and tissue regeneration, with polysaccharides and their derivatives complexed with silver being representative examples [89]. However, such polymers also have limitations. For instance, a study reported that only three out of six synthesized polymers exhibited biocompatibility [39]. Another investigation found that dextran-polyacrylamide (D-PAA) Ag/D-PAA composite materials may potentially induce erythrocyte apoptosis via ROS and Ca^2+^-mediated pathways under certain conditions. Since erythrocyte-related indices are often considered sensitive indicators for evaluating the hemocompatibility of nanomaterials, this finding suggests potential risks to the erythrocytes [90].

#### 3.4.3. Unique Physical and Chemical Properties

Silver-based polymers can also exhibit physicochemical characteristics suitable for functional expansion. When silver is incorporated into polyvinylpyrrolidone (PVP)-based hydrogel systems, increasing silver content leads to a decrease in hydrogel density from 0.6669 g/cm^3^ to 0.2963 g/cm^3^, an increase in porosity from 4% to 11.04%, and a rise in surface roughness (Ra) from 8.42 µm to 16.33 µm. These changes are expected to provide a more favorable microstructural foundation for substance exchange and cell adhesion. Meanwhile, the water vapor transmission rate (WVTR) significantly increases from 65.169 g/m^2^·h to 93.772 g/m^2^·h, indicating enhanced moisture permeability. This suggests an ability to maintain a wound environment that is moist but not excessively exudative, potentially leading to more effective management of wound exudate [91].

Furthermore, by adjusting the distance between AgNPs and photosensitizer (PS) molecules through thin layers of different Pluronic copolymers, this system achieves distance controllability and aqueous stability. It enables tunable enhancement of fluorescence emission and singlet oxygen generation for specific PS molecules, which can be further developed for theranostic applications in photodynamic therapy [92].

Under ultraviolet irradiation, Ag-Cy-PPG transforms into a water-soluble crosslinked nanogel via the formation of photo-polymerized cytosine-Ag-cytosine crosslinks, exhibiting a series of unique chemophysical properties. These include strong and stable fluorescence behavior, highly sensitive pH responsiveness, switchable phase transition behavior, and the ability to precisely control the release of silver ions (Ag^+^) in weakly acidic aqueous solutions [93].

Silver-based composites integrate functional materials to preserve silver’s potent antimicrobial properties while enhancing multifunctional capabilities, such as cell proliferation and resistance prevention. Certain composites further amplify antibacterial efficacy through synergistic mechanisms, simultaneously improving material stability and biosafety. Although numerous silver-based composites demonstrate exceptional performance in laboratory or model systems, their ultimate clinical value must be validated in real-world medical settings. Future research should prioritize evaluating these materials under complex physiological conditions—assessing antimicrobial efficiency, biocompatibility, and long-term safety—to facilitate their translation from bench to bedside.

## 4. Current Clinical Application

Some researchers suggest that selective, time-limited application of silver-based treatments can achieve dual benefits: potent antimicrobial efficacy coupled with enhanced wound healing quality, while maintaining favorable cost-effectiveness [94]. In clinical practice, silver formulation applications are tailored intrinsically to the wound type. Chronic wounds (e.g., diabetic foot ulcers) depend on silver’s sustained antimicrobial and anti-biofilm properties to address persistent inflammation and elevated infection risks [95]. Burn wounds leverage silver’s potent bactericidal capacity for infection prevention alongside its distinctive exudate control function [96]. Acute traumatic wounds (such as surgical incisions) utilize localized silver intervention to reduce secondary infection rates and accelerate healing [97]. Current optimization strategies focus on formulation selection, release kinetics, and synergistic approaches calibrated to microenvironmental characteristics and pathological demands. However, therapeutic efficacy and safety profiles require fine-tuned regulation based on wound progression stage, infection severity, and host response dynamics. Despite continuous progress in in vitro and animal model studies, the clinical application of silver-based formulations remains relatively conservative, with their therapeutic efficacy and scope of application constrained by multiple factors.

### 4.1. Chronic Wound

Chronic wounds represent the predominant application domain for silver-based materials in clinical practice. While silver-containing dressings were historically regarded as a cornerstone intervention for chronic wound infections, their preferential use has faced increasing scrutiny in recent years. Substantial evidence suggests that although silver-based products offer short-term advantages in specific scenarios, their overall therapeutic profile and safety do not demonstrate significant superiority over advanced dressing alternatives or combination therapies. Moreover, they exhibit notable limitations in achieving key clinical endpoints such as complete healing rates and scar formation incidence [98]. Meta-analyses indicate that silver-based dressings enhance chronic wound healing rates (OR: 1.43) and reduce healing time (MD: −0.96 weeks), yet demonstrate no significant effect on wound surface area reduction (MD: 12.41) [99]. On the one hand, various clinical trials have yielded inconsistent conclusions regarding the efficacy of silver dressings in reducing the area of chronic wounds [100,101]. On the other hand, the observed clinical outcome of wound healing—complete re-epithelialization—is primarily influenced by the degree of wound infection, inflammation levels, and cellular proliferation [102]. In contrast, wound area reduction relies more on fibroblast-mediated contraction. Therefore, this research suggests that silver dressings may exert their effects by inhibiting infection and alleviating inflammation, among other mechanisms [103]. In venous leg ulcers (VLUs), silver-containing dressings accelerate early healing but show comparable long-term complete healing rates to conventional dressings [104]. Comparative trials reveal superior efficacy for octenidine-hyaluronic composite dressings, with approximately 20% faster wound closure rates. Octenidine-hyaluronic composite dressings emerge as alternative options for infected, refractory wounds due to their combined low cytotoxicity and anti-biofilm activity [105,106].

Notably, the antimicrobial advantages of silver-based products face significant challenges. While negative pressure wound therapy (NPWT) combined with Ag^+^ synergistically inhibits Pseudomonas aeruginosa colonization, its overall healing efficacy remains comparable to NPWT alone [107]. Furthermore, biological combination dressings demonstrate superior bacterial load reduction compared to silver-containing hydrofiber dressings [108]. Additional concerns arise from unquantified clinical risks: commercially available products lack standardized evaluation of Ag^+^ release concentrations relative to cytotoxicity thresholds, with prolonged use potentially increasing the risk of local silver accumulation. Particularly in low-infection-risk dry wounds, the risk-benefit balance between antimicrobial benefits and potential tissue damage remains questionable [109]. More critically, silver-based dressings exhibit significant limitations in their indicated applications. Clinical studies confirm that they provide no improvement in healing rates or reduction in antibiotic usage for acute diabetic foot ulcers (*p* > 0.05) [110]. Furthermore, evidence supporting their efficacy against chronic wound biofilms remains weak, characterized by low translational success from in vitro to human studies, with insufficient data to justify a priority recommendation in this domain. Contemporary guidelines predominantly base their recommendations on short-term efficacy data while lacking assessment of long-term patient outcomes (e.g., recurrence rates, scar formation) and health economic metrics [111].

Current evidence warrants the tiered application of silver-containing products in chronic wound management. For exudative ulcers with a high infection burden (e.g., wounds colonized by Pseudomonas aeruginosa), silver dressings may serve as transitional interventions. However, in most chronic wounds—particularly those with ischemia or a low infection risk—their marginal therapeutic advantages fail to justify the cost–benefit calculus, warranting prioritized consideration of bioactive dressings (e.g., suberylamine-hyaluronic acid formulations) or combination therapies involving mechanical debridement.

### 4.2. Burns

Silver has a long-established history in burn care, with 1% silver sulfadiazine (SSD) maintaining status as the standard conservative therapy for burn wounds for over four decades [26]. However, the emerging literature from the past decade has documented significant limitations of SSD, prompting recent clinical evaluation of novel silver formulations, including silver nanoparticles (AgNPs), silver-impregnated hydrofiber dressings, and silver-impregnated foam dressings [112,113]. Contemporary burn management primarily utilizes three categories of silver-based products: SSD, AgNP dressings, and Ag^+^-supplemented materials.

Current evidence indicates silver-containing dressings demonstrate positive outcomes in exudate management, wound progression, and infection risk reduction. Lower-quality evidence suggests that superior exudate control versus iodine-based dressings (supported by 66.7% of studies), while Aquacel-Ag^®^ significantly reduces healing time and inflammatory responses compared to traditional silver sulfadiazine (SSD). However, long-term safety requires further validation [114,115]. Economic evaluations reveal Mepilex Ag™ delivers enhanced cost-effectiveness in pediatric partial-thickness burns (TBSA ≤ 10%), establishing it as a primary evidence-based recommendation [116].

Compared to traditional silver formulations, AgNP technology demonstrates multiple advantages in burn wound management. Relative to silver sulfadiazine (SSD), AgNPs not only accelerate healing (reducing time by approximately 3–5 days) but also significantly decrease dressing change frequency (The mean (standard deviation, SD) number of dressing changes in the nanocrystalline silver group was 4.1 (2.3), and the corresponding estimate in the 1% silver sulfadiazine group was 9.6 (6.7); mean difference of −5.56 (95% CI), −7.57 to −3.55, *p* < 0.001)) and improve pain control (30% reduction in VAS scores, *p* < 0.001) [117]. Clinical trials reveal that AgNP dressings reduce hospitalization duration by 15% (*p* = 0.027) compared to silver-impregnated hydrofiber dressings, while achieving 94.1% healing rates [118]. Although no statistical differences emerged in infection rates (mean 7.8% vs. 9.2%) or need for surgical intervention, AgNP groups exhibited 42% lower composite adverse event incidence, indicating potential safety benefits. Current clinical debates center on mechanistic variations among silver carriers and long-term outcomes. While most studies support AgNP advantages in healing acceleration, pain management, and hospitalization reduction, SSD retains therapeutic value in specific contexts such as high-exudate wounds and cost containment scenarios [24].

Furthermore, the combined application of silver-based products with other therapeutics demonstrates significant research value in the management of burn wounds. For partial-thickness burns, AgNP dressings combined with recombinant human epidermal growth factor (rhEGF) have been shown to reduce mean wound healing time by 5.68 days (95% CI −7.38 to −3.99, *p* < 0.00001), improve healing rates (RR 0.34, 95% CI 0.23–0.48, *p* < 0.00001), decrease scar hyperplasia incidence (RR 0.67, 95% CI 0.54–0.84, *p* = 0.0004), lower bacterial positivity rates (RR 0.50, 95% CI 0.28–0.89, *p* = 0.02), and reduce adverse reactions (RR 0.31, 95% CI 0.16–0.58, *p* = 0.0003) [119].

Thus, despite the widespread use of silver formulations in burn wound management, persistent controversies remain regarding mechanistic differences among silver carriers, long-term safety concerns, and optimal indication selection. While traditional silver sulfadiazine (SSD) retains advantages in resource-limited settings or high-exudate wounds, broader adoption of novel silver-based formulations requires further high-quality clinical evidence.

### 4.3. Acute Wound

In the management of acute wounds (e.g., surgical incisions, acute trauma), the clinical application of silver-based products remains significantly controversial and carries a low recommendation grade. International guidelines explicitly recommend prioritizing “moist wound healing” principles for acute wound management, with intervention strategies tailored to the level of contamination. For clean or minimally contaminated acute wounds, conventional dressings sufficiently maintain optimal wound microenvironment balance, whereas silver-based products lack high-grade evidence supporting their use and may potentially disrupt normal cellular proliferation through Ag^+^ release [120]. Silver-containing dressings should be reserved as short-term (typically ≤2 weeks) adjunctive antimicrobial therapy only when clear high-infection risks exist, or early biofilm formation is evident. Furthermore, clinical studies demonstrate that silver dressings fail to achieve statistically significant improvements in key acute wound healing endpoints, with significantly inferior cost-effectiveness ratios compared to conventional dressings [121].

Despite the diverse and innovative delivery methods demonstrated in research frontiers—many of which have been successfully validated in vitro and in animal models—their translation into clinical practice remains limited, with the use of silver-based products across medical fields remaining predominantly conservative. Consequently, we aim to conduct an in-depth exploration of the underlying reasons for this discrepancy and identify the key factors constraining the practical application and advancement of novel silver-based technologies.

## 5. Discussion

### 5.1. Limitations

Although silver has become the most widely used antimicrobial agent besides antibiotics in infection control, its development and practical application still face significant limitations—including the inherent constraints of silver itself and challenges in clinical implementation.

#### 5.1.1. Toxicity

While silver demonstrates relative safety at controlled concentrations, its potential toxicity warrants careful consideration. Prolonged or high-concentration exposure leads to excessive silver accumulation, inducing oxidative stress, genetic damage, and alterations in muscle protein profiles and histological structures [122]. A study revealed silver’s mean residence time (MRT) in murine blood is 135 h with an average half-life of 94 h [123]. Beyond the bloodstream, metallic nanoparticles can also translocate and accumulate in other vital organs, where they may persist for periods extending up to or even exceeding six months [124]. Regardless of the exposure route, AgNPs can cross the blood–brain barrier and progressively accumulate in brain tissue over time [36]. The particle-specific effects of AgNPs may induce mitochondrial damage and apoptosis, ultimately leading to neurotoxicity [125]. A small amount of Ag^+^ released from silver nanoparticles can trigger an influx of Na^+^ and Ca^2+^, leading to plasma membrane depolarization. This results in intracellular sodium and calcium overload, leading to cellular necrosis and eventually manifesting as tissue necrosis and inflammation [25]. For instance, exposure to a silver-containing composite at a concentration of 5 mg/L caused a significant increase in both reactive oxygen species (ROS) levels and calcium ion levels within red blood cells. Both indicators showed statistically significant elevation (*p* < 0.0001), suggesting elevated intracellular calcium levels and indicating that high concentrations of silver may induce erythrocyte death [90].

#### 5.1.2. Instability

The antimicrobial efficacy of silver predominantly depends on the release kinetics and concentration of Ag^+^ ions. Significant variations in antibacterial performance exist among different silver-based materials due to their distinct Ag^+^ release profiles, which typically diminish over time, resulting in gradual attenuation of antimicrobial activity [126]. For instance, the antibacterial effectiveness of silver nanoparticles or silver coatings in medical devices or wound dressings decreases as Ag^+^ reservoirs become depleted [127]. Consequently, designing silver-based antimicrobial materials requires careful balancing between immediate high efficacy and sustained long-term activity. Furthermore, Ag^+^ release rates and antimicrobial performance are significantly influenced by environmental conditions—particularly pH, temperature, humidity, and most notably, light exposure [128]. Under illumination, silver may undergo oxidation or other chemical reactions that compromise its antimicrobial stability, potentially leading to reduced infection control capacity.

#### 5.1.3. Drug Resistance

The issue of bacterial resistance to silver warrants significant concern, as microorganisms employ diverse and complex mechanisms to develop tolerance to silver. Certain bacteria produce adhesive flagellin proteins that induce nanoparticle aggregation, while others express efflux pumps to expel Ag^+^ ions from cells, thereby reducing toxicity actively. Some strains biochemically reduce toxic Ag^+^ to non-toxic metallic silver particles, and many activate damage repair systems to counteract silver-induced cellular injury. Notably, particular bacterial species have evolved direct inhibition strategies to minimize silver contact and subsequent damage [12,129,130]. Although clinical isolates currently exhibit low-level silver resistance and no severe resistance issues have emerged in wound care, limited research data indicate potential risks. Existing evidence demonstrates that silver resistance primarily originates from horizontal gene transfer and adaptive mutations in stress-response genes, with the widespread use of silver-based formulations in healthcare settings potentially accelerating this process. Therefore, elucidating the mechanistic differences between AgNPs and ionic silver, clarifying potential silver resistance pathways, and establishing dynamic resistance monitoring systems hold significant scientific value for optimizing the clinical application of silver-based antimicrobials and mitigating resistance development.

#### 5.1.4. Lack of Clinical Evidence

Although hundreds of novel silver-based composite dressings demonstrating superior antibacterial efficacy and unique physicochemical properties have been demonstrated at the laboratory level, very few have transitioned into clinical applications. A search on ClinicalTrials.gov reveals a striking scarcity of trials related to silver-containing materials and wound healing—fewer than 50 entries for burns, fewer than 10 for chronic wounds, and fewer than 100 for other wound types. This highlights a significant translational barrier, with most research confined to in vitro studies. At the same time, in vivo experiments and human clinical trials remain exceptionally rare, posing a significant challenge to the advancement of silver-based antimicrobial agents (Table 2).

One major obstacle in conducting clinical trials is the lack of standardized endpoints and heterogeneous evaluation metrics across studies, which often include re-epithelialization rates, patient pain scores, infection incidence, scar characteristics, and treatment costs [131,132,133]. Crucially, these parameters frequently fail to directly correlate with infection diagnosis and therapeutic efficacy, compromising data quality and overall reliability. This absence of unified assessment criteria significantly impedes robust evaluation and widespread adoption of silver-based antimicrobials. Consequently, establishing standardized clinical evaluation frameworks is urgently needed to enhance both research quality and translational potential of silver-based agents. The other critical barrier to clinical trials lies in the poor comparability among silver-based dressings, severely compromising evidence synthesis and evaluation. Substantial variations in Ag^+^ loading/release behaviors across material carriers prevent standardized dose–response establishment, while divergent antimicrobial mechanisms between silver forms (e.g., AgNPs vs. ionic silver) complicate efficacy assessment. Furthermore, heterogeneous release kinetics and microenvironment-dependent interactions lead to inconsistent therapeutic outcomes, undermining reliable comparisons of antibacterial efficacy and safety. This standardization deficit fundamentally hinders the generation of high-quality evidence and its clinical translation.

**Table 2:** Current status of clinical trials related to silver products.

Number	Current Condition	Disease Categories	Treatments	Sample Size	Index	Conclusion	Last Update Time
NCT04582045	Completed.	Acute wound-surgical infection.	Silver impregnated dressing—standard border wound bandage.	1064	Rate of postoperative infection within 30 days from surgery.	Unpublished.	19 January 2023
NCT02267122	Completed.	Acute wound-surgical infection.	Ionic silver-containing dressing—Mupirocin ointment—Conventional dressing.	147	Rate of post operative infection within 30 days from surgery.	Topical application of mupirocin ointment achieves better results for the prevention of SSI than ionic silver-containing dressing or standard dressing in patients undergoing elective open colorectal surgery [134].	17 October 2014
NCT02288884	Completed.	Acute-surgical infection.	Silver containing dressing—Standard dressing.	100	Wound complication within 6 weeks.	Unpublished.	13 October 2016
NCT01143883	Completed.	Acute-surgical infection.	Silverlon Dressing—Standard of Care Dressing.	110	Surgical Site Infection within 30 days.	Silver nylon is safe and effective in preventing surgical site infection following colorectal surgery [135].	13 June 2013
NCT01229358	Completed.	Acute-surgical infection.	Silver Eluting Dressing—Standard Gauze.	500	Wound complication rate within 30 days.	Under the study conditions, a silver-eluting alginate dressing showed no effect on the incidence of wound complications [136].	12 January 2016
NCT00981110	Completed.	Acute-surgical infection.	Mepore Self-adhesive absorbent dressing—AQUAGEL Ag Hydrofiber Wound Dressing.	120	The rate of patients with a Surgical site infection within 30 days.	This randomized trial did not confirm a statistically significant superiority of Aquacel Ag Hydrofiber dressing in reducing surgical-site infection after elective colorectal cancer surgery [101].	11 September 2012
NCT01111695	Completed.	Chronic wound—arterial or venous insufficiency.	Honey-ionic silver dressing.	30	Granulation and/or epithelial tissue progression.	Unpublished.	30 September 2011
NCT05009576	Completed.	Chronic wound.	Vac with silver—simple VAC without silver alginate.	62	1. Granulation tissue. 2. Size of wound.	Unpublished.	17 August 2021
NCT05667831	Completed.	Chronic—Pressure Injury.	Alginate silver dressing—traditional dressing.	160	1. Bacterial colony count. 2. White blood cell count. 3. High-sensitivity C- C-reactive protein.	Unpublished.	29 December 2022
NCT05824026	Completed.	Burn.	Gelling fiber wound dressing with silver.	52	Percentage of wounds healed within 14 days. Complete healing is defined as ≥95% reepithelialization.	Unpublished.	29 May 2024
NCT01439074	Completed.	Burn.	Mepilex Ag(Absorbent foam silver dressing)—Silver Sulphadiazine Ag cream.	162	Time to Healing.	Unpublished.	18 December 2017
NCT02108535	Completed.	Burn.	Nanocrystalline silver—Silver Sulfadiazine.	100	Proportion of complete epithelialization of the wound.	No evidence of a difference between nanocrystalline silver and 1% silver sulfadiazine dressings regarding efficacy and safety outcomes [24].	18 May 2021
NCT01553708	Completed.	Burn.	Epidermal growth factor with silver sulfadiazine cream—Silver zinc sulfadiazine cream.	34	Time (days)for complete epithelialization.	Unpublished.	25 March 2013
NCT01598493	Completed.	Burn.	Silver sulfadiazine cream—Activated carbon fiber impregnated with silver particles.	30	The healing rate, healing rate healed area/the number of healing days.	Unpublished.	13 July 2017
NCT02109718	Completed.	Burn.	Open Dressings with Petrolatum Jelly—Silver Sulfadiazine Gauze Dressing Group.	50	1. Number of days to complete re-epithelialization. 2. Incidence of wound infection. 3. Incidence of adverse reactions, including allergic contact dermatitis (ACD).	Petrolatum gel without top dressings may be at least as effective as silver sulfadiazine gauze dressings with regard to time to re-epithelialization, and incidence of infection and allergic contact dermatitis [137].	10 April 2014

### 5.2. Research Prospects

Silver-based approaches now still face significant challenges—notably Ag^+^ accumulation from prolonged use, potential toxicological effects, and emerging resistance issues. Consequently, future advancements in silver-based anti-infection strategies will likely focus on the following key directions.

First, technological advancement and material innovation are pivotal. With progress in nanotechnology, silver nanoparticles (AgNPs) can achieve targeted pathogen- or infection site-specific release through delivery systems or surface modifications, enhancing antibacterial efficacy while minimizing damage to healthy tissues. The integration of nanotechnology enables the development of light- and temperature-responsive dressings that dynamically regulate Ag^+^ release kinetics in response to external stimuli (e.g., light exposure, body temperature), achieving “on-demand” antibacterial effects and reducing cytotoxicity resulting from excessive release. For instance, light-responsive AgNP dressings can activate antibacterial functions under UV irradiation, making them suitable for low-exudate chronic wounds.

Second, the combined application of silver with other antimicrobial agents represents a critical future research direction. By leveraging synergistic interactions between multiple antimicrobial mechanisms, this approach can enhance antibacterial efficacy while simultaneously reducing both the risk of resistance development and individual agent dosages to minimize side effects. Such diversified antimicrobial strategies will prove particularly valuable for addressing complex infections, especially those involving multidrug-resistant pathogens.

Furthermore, the potential toxicity of silver requires continued investigation. Future research will focus on developing silver-based materials with enhanced biocompatibility or biodegradability to minimize Ag^+^ accumulation in the body and reduce long-term adverse effects. Concurrently, optimizing silver dosage and release kinetics will further improve safety profiles. A critical challenge lies in maintaining the minimum effective concentration while prolonging the duration of efficacy per application—an approach that would simultaneously reduce silver’s cytotoxic effects on wound tissue and minimize mechanical trauma from frequent dressing changes, ultimately achieving low-toxicity yet sustained antimicrobial performance. Concurrently, research on bacterial resistance mechanisms to silver warrants rigorous investigation. Genomic analyses elucidating regulatory networks of resistance-associated genes will inform optimized deployment strategies for silver-based antimicrobials while providing theoretical foundations for novel material design.

The successful clinical translation of silver-based antimicrobial products remains pivotal alongside advancements in biotechnology and materials science. To overcome existing translational barriers, a multidimensional strategy must be implemented to restructure the development pathway. First, standardized clinical trials should be established with validated efficacy assessment systems that incorporate unified endpoint evaluation metrics across three domains: microbiological evidence, clinical evidence, and laboratory evidence. Second, research should prioritize unmet clinical needs, particularly high-probability applications such as drug-resistant infections, diabetic foot ulcers, and the management of burn wounds. Additionally, clinical practice standardization is essential—requiring evidence-based guidelines for silver-based product/device utilization, reinforced market supervision, and enhanced regulatory frameworks that refine biocompatibility standards and toxicity evaluation protocols.

Silver holds significant value as an antimicrobial material in various applications, including infection control, medical devices, and wound care. Its broad potential is particularly evident in addressing antibiotic resistance challenges. With continuous advancements in nanotechnology and biomaterials science, silver’s antibacterial properties and clinical utility are expected to continue expanding. Moving forward, precision-guided and combination-based strategies promise to amplify silver’s role in healthcare, offering novel solutions for antimicrobial therapy.

## Figures and Tables

**Table 1:** Current classification and characteristics of delivery forms for silver products.

Delivery Form	Mechanism of Action	Advantages	Disadvantages	Clinical Applications	Representative Products
Silver Salts	Release Ag^+^ to directly kill bacteria	Low cost [24]; simple preparation	Low stability; prone to inducing resistance with prolonged use; silver accumulation cytotoxicity risk [25]	Widely used in treatment of burns [26]; combined with antibiotics [27]	Silver sulfadiazine cream [24]
Silver-Coupled Electrodes	Electric field-stimulated Ag^+^ release; interferes with bacterial bioelectrical effects [28]	Strong biofilm penetration [29]	Requires conductive medium; long-term safety requires validation [30]	Chronic wounds; multidrug-resistant infections [31]	Bacterial cellulose-based graphene oxide-silver nanoparticles antibacterial dressing [32]
AgNPs	Directly damage bacterial cell membranes [33]; causes oxidative stress [34]	Strong activity; high plasticity [35]	Poor stability; potential systemic toxicity [36]	Implant antibacterial coatings [37]	Porous silver coating [34]
Silver-Based polymers	Synergistic action with other materials	Multifunctionality; less resistance [38]	Complex preparation process; biocompatibility of some materials requires validation [39]	Most remain at the laboratory stage	Human Hair Keratin/PEO/PVA Nanofibers [40]

## Data Availability

No new data were created or analyzed in this study. Data sharing is not applicable to this article.
